# Extensive Comparative Genomic Analysis of *Enterococcus faecalis* and *Enterococcus faecium* Reveals a Direct Association between the Absence of CRISPR–Cas Systems, the Presence of Anti-Endonuclease (ardA) and the Acquisition of Vancomycin Resistance in *E. faecium*

**DOI:** 10.3390/microorganisms9061118

**Published:** 2021-05-21

**Authors:** Kodjovi D. Mlaga, Vincent Garcia, Philippe Colson, Raymond Ruimy, Jean-Marc Rolain, Seydina M. Diene

**Affiliations:** 1 Aix Marseille University, IRD, APHM, MEPHI, IHU-Mediterranee Infection, 13005 Marseille, France; mlagadodji@gmail.com (K.D.M.); vincent.garcia13@sfr.fr (V.G.); philippe.colson@univ-amu.fr (P.C.); jean-marc.rolain@univ-amu.fr (J.-M.R.); 2 IHU-Mediterranee Infection, Aix-Marseille University, 13005 Marseille, France; 3 Department of Bacteriology at Nice Academic Hospital, Nice Medical University, 06003 Nice, France; ruimy.r@chu-nice.fr

**Keywords:** comparative genomics, *E. faecalis*, *E. faecium*, CRISPR–Cas, vancomycin resistance, recombinations

## Abstract

Here, we performed a comparative genomic analysis of all available genomes of *E. faecalis* (*n* = 1591) and *E. faecium* (*n* = 1981) and investigated the association between the presence or absence of CRISPR-Cas systems, endonuclease/anti-endonuclease systems and the acquisition of antimicrobial resistance, especially vancomycin resistance genes. Most of the analysed Enterococci were isolated from humans and less than 14% of them were from foods and animals. We analysed and detected CRISPR–Cas systems in 75.36% of *E. faecalis* genomes and only 4.89% of *E. faecium* genomes with a significant difference (*p*-value < 10^−5^). We found a negative correlation between the number of CRISPR–Cas systems and genome size (r = −0.397, *p*-value < 10^−5^) and a positive correlation between the genome %GC content and the number of CRISPR–Cas systems (r = 0.215, *p*-value < 10^−5^). Our findings showed that the presence of the anti-endonuclease *ardA* gene may explain the decrease in the number of CRISPR–Cas systems in *E. faecium*, known to deactivate the endonucleases’ protective activities and enable the *E. faecium* genome to be versatile in acquiring mobile genetic elements, including carriers of antimicrobial resistance genes, especially *vanB*. Most importantly, we observed that there was a direct association between the absence of CRISPR–Cas, the presence of the anti-CRISPR *ardA* gene and the acquisition of vancomycin resistance genes.

## 1. Introduction

Enterococci are an ancient genus of *Enterococcaceae* that have adapted to living in complex environments and surviving in harsh conditions [1,2]. The genus *Enterococcus* comprises 54 species that are ubiquitously present in the gastrointestinal tracts of animals, including mammals, reptiles, birds and insects, which are thought to be the largest reservoir of Enterococci [3]. Two species, *Enterococcus faecalis* and *Enterococcus faecium*, are the leading cause of the vast majority of hospital-acquired Enterococci infections in humans [4]. The plasticity of the *Enterococcus* genomes allows them to rapidly respond and adapt to the environment by acquiring genetic determinants. It increases their ability to colonise and infect their host and cause diseases [3]. The success of *E. faecium* and *E. faecalis* in evolving as multi-resistant nosocomial pathogens is associated with their capacity to harbour and spray adaptive genetic materials, including antimicrobial resistance genes encoded by mobile genetic elements (MGEs) [5]. However, *E. faecium* is intrinsically more frequently reported as being more resistant to antibiotics—especially to vancomycin—than *E. faecalis* is (8.8% vs. 1.0% in Europe, 79.4% vs. 8.5% in the US and 22.4% vs. 0.1% in Canada) [6], and *vanA* and *vanB* are the most common mobile genes involved [7,8,9,10]. A sequence analysis of *E. faecalis* V583 revealed that 26% of the 3.36-Mb genome consisted of mobile elements, including 7 putative phages, 38 insertion elements and remnants of 3 integrated plasmids, as well as 3 independently replicating plasmids [4,11]. Enterococci genomic evolution has always been associated with the acquisition of vancomycin resistance genes carried by plasmids [12,13,14,15,16,17] and virulence genes [18,19,20,21]. The presence or absence of clustered, regularly interspaced, short palindromic repeats (CRISPR) can contribute to the regulation of this phenomenon and provide bacterial and archaeal immunity against subsequent invasion by plasmids and phages [22]. It is also known that restriction–modification systems and anti-endonuclease (*ardA*) play a significant role in the regulation of MGE transfer in the *Enterococcus* genus [11] and the acquisition and spread of antimicrobial resistance genes [23]. The aim of this study was to investigate the presence of recombination sites in both *E. faecalis* and *E. faecium* as evidence of MGE transfer and the association between the absence or presence of a CRISPR–Cas system, an endonuclease and anti-endonuclease system and the acquisition of antimicrobial resistance genes—especially the vancomycin resistance genes *vanA*, *vanB* and *vanC*—using sequenced genomes of *E. faecalis* from Marseille and publicly available genomes of both species.

## 2. Materials and Methods

### 2.1. Whole Genome Sequencing and Sequence Extraction from NCBI

We sequenced six genomes of *E. faecalis* composed of four strains isolated from humans and two from chicken faeces. The sequencing was initiated from the fact that an initial MALDI-TOF spectrum analysis revealed that they were closely related. We conducted whole genome nucleic acid extraction from the six strains using the QIAGEN automated method. We sequenced the *E. faecalis* genome using MiSeq Technology (Illumina Inc., San Diego, CA, USA) with the mate pair strategy [24]. The reads were assembled using A5-Miseq [25]. The scaffolds were re-ordered and aligned against a reference genome, *E. faecalis* ATCC 22809, using the Mauve aligner (v2.3.1) [26] with default parameters. For the purpose of the extensive comparative analysis, we extracted a total of 1591 whole genome sequences of *E. faecalis* and 1981 of *E. faecium* from the NCBI database. We used the ncbi-genome-download command with default parameters to download all available genomes (in fasta format) of *E. faecalis* and *E. faecium*, including their metadata. Only released genome sequences available for downloading were retrieved. Then, we thoroughly checked the metadata and excluded all the known contaminated sequences. Generally, the *E. faecalis* and *E. faecium* draft genome size is between 2.2 and 3.5 Mb (including plasmid sequences). In our analysis, whole genome sequences above 4 Mb with a percentage of aligned sequences (mauve_Aligner) < 50% were also excluded from our analysis. We re-annotated all 3572 genomes of the two species, including genomes sequenced from Marseille (strains G823, G824, G881, G882, G883 and G884 with assembly accession numbers FPDY01, FPDW01, FPEB01, FPDZ01, FPEC01 and FPEA01, respectively), with Prokka [27] using Pfam and the Swissport database and default parameters.

### 2.2. Genome Phylogenetic Tree Reconstruction of Both E. faecalis and E. faecium

We performed a whole genome SNP alignment of the 1591 whole genomes of *E. faecalis* and the 1981 genomes of *E. faecium* using Scapper (https://github.com/tseemann/scapper, accessed on 10 January 2021), with default settings, to reconstruct the whole genome phylogenetic tree. The strains of the *E. faecalis* ATCC 29212 (NZ_CP008816.1) and *E. faecium* DO (NC_017960.1) genomes were used as references, respectively. Aligned multi-fasta were shrunk using SNP-sites [28] to remove the monomorphic sites, and so reduce the phylogenetic inference computing time. We inferred approximately maximum likelihood phylogenetic trees from shrunken alignments of nucleotide sequences using RaxML [29,30,31] and used them to adjust the seed parameter to 2000 for a reproducible and consistent tree topology. The generated phylogenetic tree was used as the entry tree for ClonalFrameML analysis [32].

### 2.3. Detection of Recombination Hotspots inside the Genomes of E. faecalis and E. faecium

We putatively identified base substitution and recombination loci containing elevated densities of base substitutions suggestive of horizontal transferring sequences. We constructed a maximum likelihood phylogenetic tree based on the putative point mutations outside these regions of high sequence diversity using ClonalFrameML (version v1.0-19) [32]. We reconstructed the evolutionary maximum likelihood phylogeny by determining genetic genealogy and considering points of variation and genome plasticity and then generated a genome recombination heatmap against phylogenetic trees.

### 2.4. Detection of Clustered, Regularly Interspaced, Short Palindromic Repeat (CRISPR) Spacers inside the E. faecalis and E. faecium Genomes

Detection of a CRISPR–Cas system (spacers, repeats) was conducted using MinCED software (v0.4.1 [33]. We set the minimum number of repeats a CRISPR–Cas system must contain to three, the minimum length of the CRISPR repeats to 23 nucleotides, the maximum length of the CRISPR repeats to 47 nucleotides, the minimum length of the CRISPR spacers to 26 nucleotides and the maximum length of the CRISPR spacers to 50 nucleotides.

### 2.5. Orthologous Gene Detection and Pan-Genome Analysis of Both Species

The *pan-genome* analysis was performed using Roary [34] (version 3.6.8) with default parameters for orthologous gene similarity (BLAST: 95% minimum identity; *E*-value = 1e^−5^), and genes had to be present in >99% of all isolates to be included in the hard-core genome. We determined core (hard-core) (genes present in 99–100% taxa), softcore (genes present in 95–99% taxa), shell (genes present in 15–95%) and cloud genes (genes present in 0–15% genomes) as described by Kaas [35]. We generated a binomial pan-genome profile for the presence, indicated as (1), and absence of genes, indicated as (0), inside the genome of both species. Orthologous genes related to antimicrobial, endonuclease and anti-endonuclease gene distribution were extracted using homemade scripts and plotted against maximum likelihood phylogenetic trees in both species.

### 2.6. Statistical Analysis

All statistical analyses conducted in this study were performed using R statistical software (v. 3.4.4) [36]. We used the Student’s *t*-test for means’ comparison, and the Pearson chi-squared test was used for proportion comparison inside and between the two species. The Pearson correlation test was used to show a statistical association between two genomic features. A logistic regression analysis was used to compute the association between qualitative genomic variables (presence and absence of vancomycin genes and the number of recombination and CRISPR spacers detected inside and across the genomes of both species). The odds ratio was calculated to interpret the association. We set the CI level to 95%. The statistical test was significant at a *p*-value of < 0.05. All *p*-values below 0.00001 were standardized as *p*-value < 10^−5^ in this study.

## 3. Results

### 3.1. Comparison of the E. faecalis and E. faecium Genome Features Reveals Differences in Genome Size and No Difference in GC Percentage

We assembled the clinical Marseille strains G823, G824, G883 and G884 into 2.89, 3.096, 2.76 and 2.95 Mb, respectively, and the animal (chicken) strains G881 and G882 into 2.97 and 3.25 Mb, respectively. Overall, as presented on Figure S1A, 34.25% (545/1591) of *E. faecalis* genomes were from North America followed by 30.48% (485/1591) from Europe, and 34.42% (682/1981) of *E. faecium* genomes were from Europe. We noted that 31.99% (509/1591) of the *E. faecalis* strains and 55.12% (1092/1981) of the *E. faecium* strains were isolated primarily from humans. However, a considerable number of *E. faecalis* (14.26%, 9.05%) and *E. faecium* (9.74%, 6.86%) were isolated from food and animals, respectively (Figure S1B). The average genome size of *E. faecalis* is estimated at 3.08 Mbp (min = 2.60 Mbp, max = 3.59 Mbp, sd = 0.15) and that of *E. faecium* at 2.91 Mbp (min = 2.23 Mbp, max = 3.72 Mbp, sd = 0.21), with a statistically significant difference between the two species (*p*-value < 10^−5^). The average DNA coding sequence size of *E. faecalis* is estimated to be 2948 (min = 2357, max = 3423, sd = 173) and that of *E. faecium* to be 2708 (min = 2098, max = 4855, sd = 235). The GC percentage of *E. faecalis* is 37.42% (min = 36.80%, max = 39.67%, sd = 0.62), while that of *E. faecium* is 37.90% (min = 37.10%, max = 39.87%, sd = 0.43), with no significant difference (*p*-value = 3.97). The overall analysis of genome features indicates a statistically significant difference between *E. faecalis* and *E. faecium*. All available metadata associated to the analysed genomes are provided in Tables S1 and S2.

### 3.2. The Genomes of E. faecalis Contain a High Density of Recombination Hotspots Compared to E. faecium

**Figure 1:**
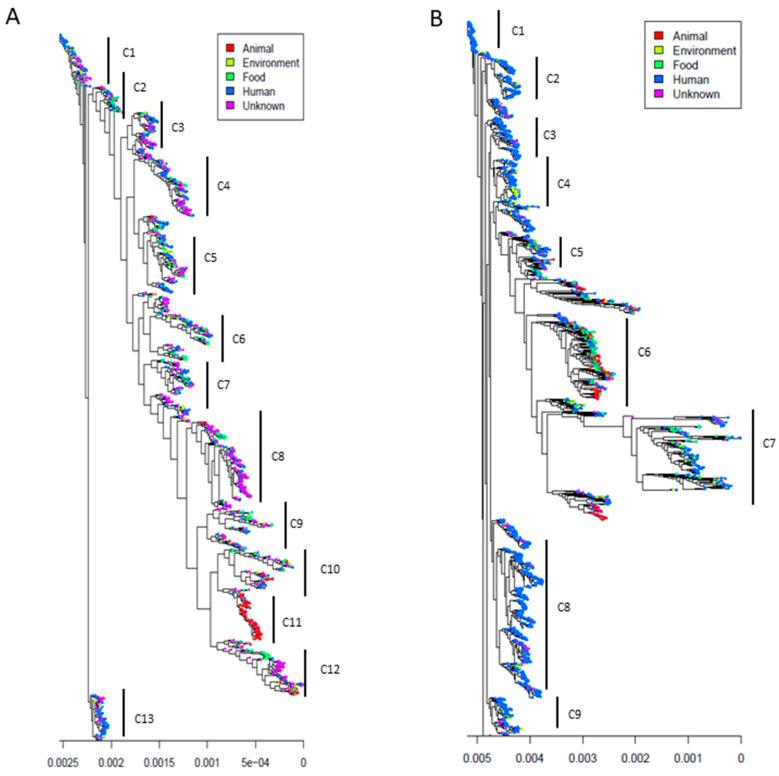
Maximum likelihood phylogenetic tree of 1591 *E. faecalis* genomes and 1981 *E.* *faecium* genomes. (**A**): We detected 13 phylogenetic clades in *E. faecalis* (left); almost all clades are heterogenous, with a mixture of *E. faecalis* from humans, animals, foods, environment and unknown sources. Two clades, namely C11 and C13, appeared specific to animals and humans, respectively. Most of the *E. faecalis* strains are distant from ancestral strains. (**B**): In *E. faecium* (right), nine phylogenetic clades were detected. Despite the fact that all clades are composed of a combination of human and non-human strains, we can observe a predominance of *E. faecium* strains from human sources. Both phylogenetic trees were generated from an SNP alignment of the whole genome sequences. Monomorphic sites were trimmed from the alignment, and a first phylogenetic tree was produced using RAxML with the generalized reverse-time method. We used these trees as entering trees for ClonalFrameML analysis. Final midpoint-rooted clonal phylogenetic trees were generated.

We processed a whole genome SNP alignment for both species. The phylogenetic tree of *E. faecalis* shows 13 different small clades defined as a minimum of 20 to 30 branches sharing a common node (Figure 1A). With the exception of clade C11, which almost exclusively contains animal strains, and clade 13, which is predominantly composed of human strains, the remaining 11 clades consist of a mixture of human, animal, food and environmental strains. The length of the phylogenetic branches indicates the level of genomic evolution from the ancestral strain. Similar observations were made with *E. faecium*, with nine small heterogenous clades (Figure 1B). As can be observed in Figure 1B, most of the *E. faecium* isolates were from humans.

**Figure 2:**
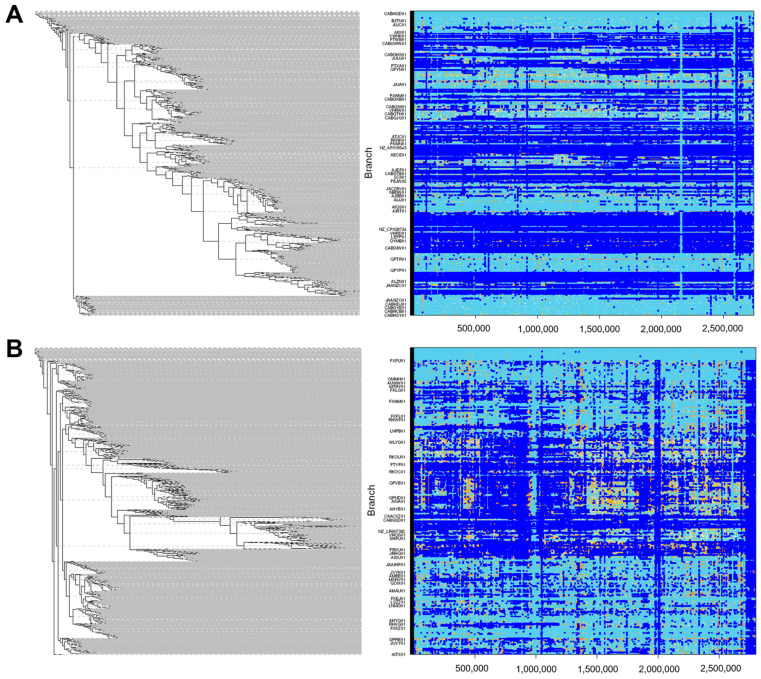
Clonal phylogeny and inferred recombination events. Midpoint-rooted clonal phylogeny (left) of 1591 *E. faecalis* isolates (**A**) and 1981 *E. faecium* isolates (**B**). Recombination events (right) were estimated as described in the Method section. The sizes and genomic locations of recombination fragments (dark-blue line segments) occurring along branches in the phylogeny are aligned with branches in the phylogeny.

We detected recombination sites in 66% (1045/1591) of the genomes of *E. faecalis* that were analysed (Figure 2A). The number of recombination blocks is proportional to the length of the phylogenetic branch, which denotes a high level of recombination among these strains. Moreover, we observed that these strains contained a substantial number of animal strains. However, we detected recombination locks in only 30.94% (613/1981) of the *E. faecium* genomes (Figure 2B). We observed a similar profile as with *E. faecalis*, where most of the recombination occurred within the clades containing a mixture of isolates (animal, environmental, food and human). Overall, there were more genomic recombination hotspots detected in *E. faecalis* than in *E. faecium*, with a statistically significant difference (*p*-value < 10^−5^). Moreover, most of these recombination hotspots occurred on branches with mixtures of strains from animals, food and the environment compared to those unique to humans.

As presented in Table S3, the pan-genome analysis identified a total of 39,665 orthologous genes in *E. faecalis* and 45,697 in *E. faecium*. Both species present a similar pan-genomic profile, with an exponential increase in the total number of orthologous genes and new genes when we added more genomes to the pan-genome. This suggests that the pan-genome of these two species is open. The hard-core genome of *E. faecalis* represents 30.73% of the average proteome, while that of *E. faecium* is estimated at 19.23%. A high number of orthologous genes were included in cloud genes (accessory genes)—36,161 and 41,885 for *E. faecalis* and *E. faecium*, respectively—suggesting a prominent level of genome plasticity.

### 3.3. There Were More CRISPR–Cas Systems and Absence of Anti-Endonuclease in E. faecalis Genomes Compared to E. faecium

We detected CRISPR–Cas systems in 75.36% (1199/1591) of *E. faecalis* genomes and in only 4.89% (97/1981) of *E. faecium*, a statistically significant difference (*p*-value < 10^−5^). In *E. faecalis*, the number of CRISPR–Cas systems detected varied from 0 to 29 and contained five on average per genome. In *E. faecium*, it varied from 0 to 21, with less than one on average per genome. We found a positive association between the number of CRISPR spacers detected and the number of recombination blocks detected in *E. faecalis* (F-statistic: 14.39, *p*-value < 10^−5^). Three CRISPR–Cas-associated coding proteins—CRISPR–Cas1, CRISPR–Cas2 and CRISPR–Cas9—were identified in both species. We detected two variants of Cas9 (Cas9 and Cas9.1) in *E. faecalis* alone (sequence identity cut-off set at 80%).

### 3.4. The Association between the Absence of CRISPR–Cas Systems, the Presence of Anti-Endonuclease Genes (ardA) and Acquisition of the Vancomycin Resistance Genes vanA, vanB in E. faecium

**Figure 3:**
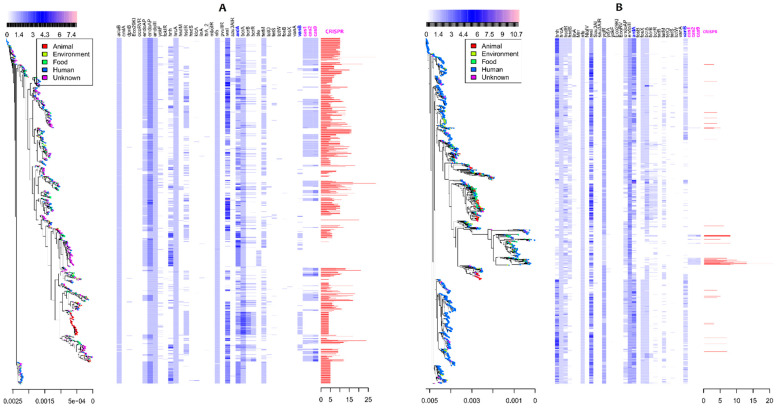
Heatmap of antimicrobial orthologous genes detected in *E. faecalis* (**A**) and *E. faecium* (**B**) plotted against a maximum likelihood phylogenetic tree. Phylogenetic tree (left). Restriction enzymes/endonucleases and antimicrobial genes’ distribution (centre), including CRISPR–Cas proteins; blue to pink ticks show the presence of orthologous genes in the corresponding genome on the phylogenetic tree; an empty space illustrates the absence of the genes. Red bars (right) show CRISPR spacers, with a scale indicating the presence and the number of spacers identified. Genes shown in the top horizontal bars are restriction enzymes/endonucleases/anti-endonucleases; 5-methylcytosine-specific restriction enzyme B (*mcrB*); anti-restriction protein (*ardA*); restriction endonuclease (*cfrBI*); restriction endonuclease (*eco29kI*); restriction-modification methylase (*eco57I*); EcoKI restriction-modification system protein (*hsdS*); 5-methylcytosine restriction system component (*mcrBC*); restriction system protein (*mrr*); restriction endonuclease (*ngoBV*); restriction endonuclease (*ngoFVII*); putative type-1 restriction enzyme specificity protein (*mg438*); putative type-1 restriction enzyme specificity protein (MPN_089); restriction endonuclease (*sinI*); Type I restriction enzyme EcoKI M protein (*hsdM*); Type I restriction enzyme EcoKI M protein (*hsdM*); Type I restriction enzyme EcoKI M protein (*hsdR*); Type I restriction enzyme (*EcoR124II*); Type II restriction endonuclease (RE_Alw26IDE); Type III restriction enzyme (Type III); Type II restriction enzyme (*sau3AI*); antimicrobial resistance genes: *bmrA* (multidrug resistance ABC transporter); *qacA* (multidrug efflux protein); *msbA* (lipid ABC transporter permease/ATPase; multidrug resistance ABC transporter); *ble* (bleomycin-resistant genes); *emrY* (multidrug resistance protein Y); *bcr* (bicyclomycin/multidrug efflux system); *yheH*, *yheI* (multidrug resistance ABC transporter); *fosB* (fosfomycin resistance gene B); *fosX* (fosfomycin resistance gene X); *tetA*, *tetC*, *tetD*, *tetM*, *tet*R (tetracycline resistance gene class A, C, D, M and R); *vanA* (vancomycin resistance gene A); *vanB* (vancomycin resistance genes B); *linA* (lincosamide B resistance genes); *cmlA* (chloramphenicol efflux protein); *cas1*, *cas2*, *cas9*, *cas9*-1 (CRISPR-associated coding protein genes).

We retrieved antimicrobial resistance genes, endonuclease genes, anti-endonuclease genes and presence and absence matrices from the overall pan-genome matrices, and the heatmaps were plotted against the maximum likelihood phylogeny trees to analyse their distribution in both *E. faecalis* (Figure 3A) and *E. faecium* (Figure 3B). We observed that the vancomycin resistance gene *vanB* was present in both species. *E. faecium* harbours more vancomycin resistance genes than *E. faecalis* does (*E. faecium*/*E. faecalis*: 1069/201, *p*-value < 10^−5^). Most importantly, we detected the presence of vancomycin resistance genes in the genomes where a CRISPR–Cas system was absent in both species. Furthermore, endonuclease genes, including *cas1*, *cas2* and *cas9*, were found in both species, with a slight increase in *E. faecium*. However, anti-endonuclease genes (*ardA*) were found in large amounts in *E. faecium*. The presence of a CRISPR–Cas system in the genome of *E. faecium* decreased by 0.97 times the acquisition of vancomycin-resistant genes (estimates = −0.972, OR = 0.68, *p*-value < 10^−5^, CI = [0.587–0.804]). The number of recombination hotspots detected in the genomes of both species decreased by 0.08 times the acquisition of vancomycin resistance genes (estimate = −0.08, OR = 0.98, CI = [0.956–0.995], *p*-value = 0.00021). Overall, there was a direct association between the absence of CRISPR spacers, the presence of anti-endonuclease genes (*ardA*) and the acquisition of vancomycin resistance in *E. faecium*.

## 4. Discussion

*E. faecalis* and *E. faecium* are the main causes of Enterococci nosocomial infections [3] and have been widely reported in blood [37] and in urinary tract infections [38]. In the past decade, the emergence of these two species has been attributed to resistance to antibiotics used in treating human infections, especially vancomycin [39]. Palmer et al. and Van Hal et al. demonstrated that vancomycin resistance genes, especially *vanA* and *vanB* and their associated regulatory genes *vanAHXZ* and *vanBYXZ*—are transferable by mobile genetic elements and plasmids [40,41]. Furthermore, Palmer et al. demonstrated that multidrug-resistant Enterococci lack the CRISPR–Cas protein in their genome [22]. Here, we performed an extensive comparative analysis of 3572 publicly available genomes of *E. faecalis* (1591) and *E. faecium* (1981), including six genomes of *E. faecalis* sequenced in Marseille. One limitation of our study can be identified regarding the fact that not all genomes were linked with their metadata; thus, there were a considerable number of genomes with missing metadata, such as the geographical origins and/or the isolation source of the strain. This includes 31.68% of the *E. faecalis* and 24% of the *E. faecium* genomes analysed in this study. Our study confirmed the high rate of vancomycin-resistant *E. faecium* reported around the world compared to *E. faecalis*, and the association with the presence or absence of a CRISPR–Cas system and its associated protein Cas in their respective genomes. The CRISPR–Cas system (spacers and related proteins) provides bacteria and archaea with a sequence-specific acquired defence system against plasmids and phage acquisition [42] and adaptive immunity against foreign elements. When the virus injects its genetic element into the bacteria, a small sequence of the viral genome, known as a spacer, is integrated into the CRISPR locus to immunize the bacteria. Spacers are transcribed into small RNAs that guide the direct cleavage of viral DNA by Cas nuclease proteins. The immunized population not only acquires resistance to its predators but also passes this resistance mechanism vertically to its progeny [42,43]. In this study, we observed that *E. faecalis* genomes contain a significantly higher number of recombination hotspots than *E. faecium* genomes do. *E. faecalis* is armed with a substantial number of CRISPR–Cas systems that protect the bacteria from acquiring subsequent external DNA, such as mobile genetic elements and plasmids. Therefore, we found a positive correlation between the number of recombination hotspots and the presence of CRISPR spacers found in *E. faecalis*. The massive *ardA* orthologues detected in *E. faecium* are evidence of the acquisition of plasmid elements, which are carriers of the vancomycin resistance genes *vanA* and *vanB* [10,39,44]. Our study also showed that there were more vancomycin resistance genes (*vanA* and/or *vanB*) detected in *E. faecium* than in *E. faecalis*, with a statistically significant difference. We found that the presence of a CRISPR–Cas system is protective for *E. faecalis* in acquiring specific mobile genetic elements carrying the vancomycin resistance genes *vanA* and *vanB*. However, the presence of *ardA* genes inactivates the function of endonuclease protective activities and enables the genome of *E. faecium* to be versatile in acquiring external DNA horizontally. The anti-endonuclease gene *ardA* is known to regulate horizontal gene transfer, causing multidrug resistance in *Enterococcus* [4,11] and actively contributing to the acquisition and dissemination of antimicrobial resistance genes [23]. These observations explain why *E. faecium* is often reported to exhibit more resistance to vancomycin than *E. faecalis* is. In this analysis, CRISPR–Cas9 was detected in *E. faecalis* as well as CRISPR–Cas1 and CRISPR–Cas2, and was almost absent in *E. faecium*. CRISPR–Cas1 and CRISPR–Cas2, two metal-dependent nucleases, are both necessary and sufficient for spacer acquisition, but dispensable for target interference [43,45]. However, CRISPR–Cas9, the sole Type II Cas protein, is involved in target surveillance and interference [46]. The endonuclease activity of Cas9 is dispensable for acquisition, as its role is to select spacers, whereas Cas1, whose non-specific nuclease activity is required for adaptation, cleaves the contiguous sequence, yielding a precisely selected spacer sequence [43]. The phylogenetic analysis revealed that some strains of *E. faecalis* and *E. faecium* isolated from humans share the same nodes with animal, food and environmental strains, suggesting that these strains may find their origin in animals and environments with zoonotic transmission. This hypothesis is supported by Lebreton et al. [39], who discovered that an epidemic hospital-adapted lineage of *E. faecium* emerged approximately 75 years ago—concomitant with the introduction of antibiotics—from a population that included mostly animal strains, rather than from human commensal lines. The overuse or misuse of antimicrobial agents in animal farming can exert selection pressure on microbial communities and select resistant bacteria. These resistant bugs can pass from the environment and foods to humans through food and freshwater consumption. Humans develop infections due to multi-resistant bacteria that become difficult to treat in therapy. This explains why *E. faecium* strains are more associated with zoonotic dissemination, and the emergence of vancomycin-resistant *E. faecium* in humans may be related to the use of avoparcin as an animal growth promoter, known to produce cross-resistance to vancomycin [47].

## 5. Conclusions

This study shows that extensive genomic recombination has occurred in the *E. faecalis* species due to mobile genetic elements and phages capable of inducing adaptive immunity with the acquisition of a CRISPR–Cas system. This protects *E. faecalis* from acquiring external DNA sequences carrying the vancomycin resistance genes *vanA* and *vanB*. It correlates with the reduced number of CRISPR–Cas systems found in *E. faecium* and the substantial number of anti-endonuclease *ardA* genes and vancomycin resistance genes found. The emergence and dissemination of *E. faecium* infection may be due to zoonotic transmission, and the misuse of antibiotics (avoparcin) may cause the selection of emerging vancomycin resistance in Enterococci. This finding explains why *E. faecium* is more reported worldwide as a vancomycin-resistant *Enterococcus* species than *E. faecalis*.

## Supplementary Materials

The following are available online at https://www.mdpi.com/article/10.3390/microorganisms9061118/s1, Figure S1: Distribution of the geographical location and source of isolation of *E. faecalis* and *E. faecium*; Tables S1 and S2: List of all analysed genomes of *E. faecalis* and *E. faecium* with their metadata. Table S3: Distribution of pan-genome component in both *E. faecalis* (*n* = 1591 genomes) and *E. faecium* (*n* = 1981 genomes).
