# Supplementary material for: Extensive Comparative Genomic Analysis of Enterococcus faecalis and Enterococcus faecium Reveals a Direct Association between the Absence of CRISPR–Cas Systems, the Presence of Anti-Endonuclease (ardA) and the Acquisition of Vancomycin Resistance in E. faecium
Source: Microorganisms. 2021 May 21;9(6):1118. doi: 10.3390/microorganisms9061118 (PMC8224324; doi:10.3390/microorganisms9061118)
Supplement: Supplementary file 1 [file microorganisms-09-01118-s001.zip › microorganisms-1211750-supplementary/supplementary_files/Supplementary files.pdf]

microorganisms

Supplementary files

Supplementary files. Table S3: Distribution of pan-genome component in both *E. faecalis* (n= 1,591 genomes) and *E. faecium* (n=1,981 genomes).

Pan-genome component	*E. faecalis*		*E. faecium*	
	**No. of genomes**	**No. of genes**	**No. of genomes**	**No. of genes**
**Total orthologous genes**	-	39,665	-	45,697
**Hard-core genes**	1,591	906	1,981	521
**Soft-core genes**	1,511 – 1,575	477	1,881 – 1,961	269
**Shell genes**	238 – 1,510	2,121	297 - 1880	3,049
**Cloud genes**	0 - 237	36,161	0 - 296	41,858

microorganisms

MDPI

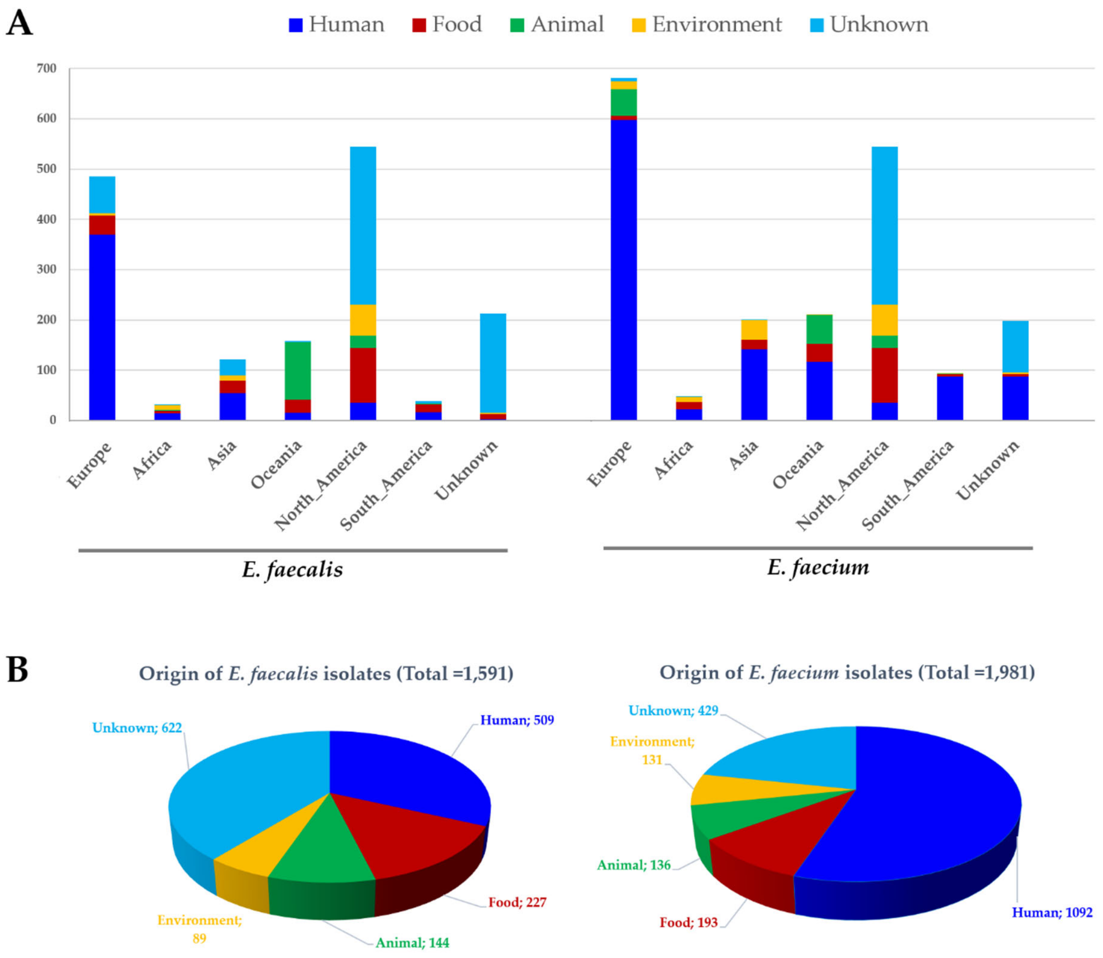

Supplementary files. Figure S1: Distribution of the geographical location and source of isolation of *E. faecalis* and *E. faecium.* (A) Origin of isolates per geographical location. Unknown refer to isolates with no metadata informations. (B) Origine of isolates based on their isolation sources.
